# Tempol Moderately Extends Survival in a hSOD1^G93A^ ALS Rat Model by Inhibiting Neuronal Cell Loss, Oxidative Damage and Levels of Non-Native hSOD1^G93A^ Forms

**DOI:** 10.1371/journal.pone.0055868

**Published:** 2013-02-06

**Authors:** Edlaine Linares, Luciana V. Seixas, Janaina N. dos Prazeres, Fernando V. L. Ladd, Aliny A. B. L. Ladd, Antonio A. Coppi, Ohara Augusto

**Affiliations:** 1 Departamento de Bioquímica, Instituto de Química, Universidade de São Paulo, São Paulo, Brazil; 2 Laboratory of Stochastic Stereology and Chemical Anatomy, Department of Surgery, College of Veterinary Medicine, University of São Paulo, São Paulo, Brazil; University of Florida, United States of America

## Abstract

Amyotrophic lateral sclerosis (ALS) is a fatal neurodegenerative disease characterized by the progressive dysfunction and death of motor neurons by mechanisms that remain unclear. Evidence indicates that oxidative mechanisms contribute to ALS pathology, but classical antioxidants have not performed well in clinical trials. Cyclic nitroxides are an alternative worth exploring because they are multifunctional antioxidants that display low toxicity in vivo. Here, we examine the effects of the cyclic nitroxide tempol (4-hydroxy-2,2,6,6-tetramethyl piperidine-1-oxyl) on ALS onset and progression in transgenic female rats over-expressing the mutant hSOD1^G93A^ . Starting at 7 weeks of age, a high dose of tempol (155 mg/day/rat) in the rat´s drinking water had marginal effects on the disease onset but decelerated disease progression and extended survival by 9 days. In addition, tempol protected spinal cord tissues as monitored by the number of neuronal cells, and the reducing capability and levels of carbonylated proteins and non-native hSOD1 forms in spinal cord homogenates. Intraperitoneal tempol (26 mg/rat, 3 times/week) extended survival by 17 days. This group of rats, however, diverted to a decelerated disease progression. Therefore, it was inconclusive whether the higher protective effect of the lower i.p. dose was due to higher tempol bioavailability, decelerated disease development or both. Collectively, the results show that tempol moderately extends the survival of ALS rats while protecting their cellular and molecular structures against damage. Thus, the results provide proof that cyclic nitroxides are alternatives worth to be further tested in animal models of ALS.

## Introduction

Amyotrophic lateral sclerosis (ALS) is a fatal neurodegenerative disease characterized by the progressive dysfunction and death of motor neurons in the motor cortex, brain stem, and spinal cord. The cause of the disease is unknown in the majority of cases, which are classified as sporadic ALS, but approximately 10% of the cases have a genetic basis. ALS-causing mutations have been identified in several genes, but the mutation of Cu,Zn superoxide dismutase gene (SOD1) is the most studied and responsible for about 20% of the familial cases [1], [2]. SOD1-linked ALS patients exhibit pathology and symptoms similar to those of sporadic ALS patients leading to the long-pursued hypothesis that there may be a common pathogenic mechanism for both forms of the disease [1]. Despite the many advances made in recent years, such a pathogenic mechanism remains under scrutiny (reviewed in 2–9).

Accumulated evidence indicates that ALS-SOD1 mutants cause motor neuron death via a toxic gain-of-function that is independent of the normal antioxidant function of the enzyme. However, the nature of the toxic gain-of-function imparted by ALS-SOD1 mutations remains elusive. More than 100 mutations distributed throughout the 153-residue SOD1 polypeptide have been shown to cause ALS; the common features of several ALS-SOD1 mutants have been characterized in vitro, such as the capability to generate oxidants, instability, a higher sensitivity to denaturing conditions and the propensity to form aggregates [2]–[9]. However, there is no single molecular feature that has been identified in all mutants, which suggests that multiple mechanisms of neuron death may operate during this disease. Non-cell autonomous mechanisms that reflect mutant SOD1-mediated damage within non-neuronal cells have also been implicated in the disease [9], [10].

The involvement of oxidative mechanisms in ALS pathology is indicated by the consistent detection of oxidative damage in DNA, proteins, and lipids within the pathologically affected areas of the patient´s central nervous system [4], [11]–[13]; these damages are reproduced in animal and cell disease models [14]–[16]. In addition to causing injury, oxidative damage may exacerbate other mechanisms that lead to neurodegeneration [4], [11], [17], [18]. Thus, oxidative damage is likely to contribute to the loss of motor neurons in ALS, independent of being the primary cause of neuron death or the secondary consequence of other toxic insults. Although antioxidants have not performed well in clinical trials [19], [20], a recent meta-analysis of drug treatment trials with the mutant SOD1^G93A^ ALS mouse model concluded that antioxidants have been the most effective drug group for extending survival among treated animals [21]. As previously discussed, the contradiction between clinical and animal studies may be partially due to the failure to achieve antioxidant concentrations in the central nervous system that are sufficient to show a therapeutic effect in patients [11].

Despite the on-going discussion about drug trials and the relevance of rodent models to human neurodegenerative diseases [21]–[24], the fatality of ALS disease justifies further attempts to identify new therapeutic leads. An alternative to be explored are cyclic nitroxides because they are multifunctional antioxidants that display low toxicity in experimental animals [25]–[30]. Tempol (4-hydroxy-2,2,6,6-tetramethyl piperidine-1-oxyl) is the most investigated cyclic nitroxide in animal disease models. Here, we examine the effects of tempol on ALS onset and progression in transgenic rats over-expressing the mutant hSOD1^G93A^, which reproduce the pathology and symptoms of ALS patients [31]. Although the results showed a modest effect of tempol in extending the survival of the animals, the association of improved animal behavior with cellular and molecular protection provided a proof of concept. Therefore, our current findings pave the way for further studies of the protective effects of cyclic nitroxides in ALS animal models.

## Materials and Methods

### Materials

All reagents were purchased from Sigma, Merck, Bayer, Roche or Fisher and were analytical grade or better. The hydroxylamine of tempol was a generous gift from Dr Vitor F. Ferreira from the Universidade Federal Fluminense, Brazil, and was synthesized as previously described [32]. All solutions were prepared with distilled water purified with a Millipore Milli-Q system.

### Ethics statement

All experiments were conducted in strict agreement with the National Institutes of Health Guidelines for the Humane Treatment of Animals and were reviewed and approved by the local Animal Care and Use Committee (Comissão de Ética em Cuidados e Uso Animal) (Permit Number: 03/2007). All surgery was performed under anesthesia, and all efforts were made to minimize animal suffering.

### Transgenic rats

Hemizygous male rats expressing the mutant human SOD1^G93A^ line 26 were obtained from Taconic in 2006 and bred with wild-type Sprague-Dawley females to establish a colony and to maintain the line [33]. Transgenic progeny were identified by detecting the exogenous hSOD1 transgene by amplification of the pup tail DNA extracted at 25 days of age using the polymerase chain reaction. The primers and cycling conditions were described previously [33]. Despite our strict adherence to the recommendations of Howland *et al*., [33], the colony has diverted and the disease progression has changed during the course of this work as has been recently recognized by Taconic on its homepage. Nevertheless, each experimental group of animals was composed of wild-type, G93A-treated and G93A-untreated littermates, which permitted the comparisons among the animals. In fact, these evolving rats may better mimic the heterogeneous genetic background of human patients.

### Animal treatment

At 7-weeks old, female rats expressing the mutant human SOD1^G93A^ (ALS animals) were randomly divided in tempol-treated and untreated littermates; wild-type littermates were used as control animals. Three treatment regimens were employed and started immediately. The first experimental group of animals received tempol (5 mM) as their drinking water *ad libitum* whereas untreated animals received water [34]. In the second experimental group, the treated animals received (30 mM) as the drinking water *ad libitum*. The animals consumed similar volumes of water or tempol solution (approximately 30 ml/day). The third experimental group of animals received tempol (26 mg in 0.5 ml saline/animal) intraperitoneally each Monday, Wednesday and Friday morning, whereas untreated animals received saline intraperitoneally.

### Disease progression

To evaluate disease progression, the body weights and the motor scores of the rats were monitored twice a week between 1 PM to 5 PM as previously described [31] with slight modifications. In summary, rats capable of maintaining their position at 80° in the inclined plane test and without symptoms scored a 5; mildly sick rats (hind or fore limb weakness and tremulous movement but a 80° position in the inclined plane test) scored a 4; moderately sick rats (dragging of a hind or fore limb, a hunched posture and a 70 to 55° position in the inclined plane test) scored a 3; sick rats (hind or fore limb paralysis and a <55° position in the inclined plane test) scored a 2; severely sick rats (difficult in movement and the incapability to sustain position in the inclined plane test) scored a 1; end-point rats (cannot correct itself from either side in 30 s) scored a 0 and were euthanized.

### Histochemistry of spinal cords

A parallel group of female G93A rats untreated (n = 5) and treated (n = 5) with 30 mM tempol in the drinking water and wild-type littermates (n = 5) was used for the stereological analysis of spinal cords. At the middle symptomatic phase of the G93A rats (115–120 days), the animals were anesthetized with ketamine (20 mg/kg) and xylazine (1.5 mg/kg) and the euthanasia conducted with an overdose of pentobarbital (100 mg/kg). The animals were perfused transcardially with phosphate-buffered saline (PBS, pH 7.4) containing 2% heparin and 0.1% sodium nitrite followed by fixative solution of 4% formaldehyde and 0.1% glutaraldehyde in PBS. The lumbar spinal cords (L1–L6) were identified, removed *en bloc* and its length measured using a digital pachymeter (Digimess). Subsequently, lumbar spinal cords were immersed in the same fixative solution for at least 72 hs, embedded in a 10% agar solution and sectioned with a nominal thickness of 40 µm using a VT1000S Leica vibratome. The sections were stained with 1% toluidine blue or used for choline acetyltransferase (ChAT) immunohistochemistry [35]. Lumbar spinal cord vibrosections for immunohistochemistry were washed with PBS, incubated with a 0.3% Triton X-100 solution, exposed to 0.3% hydrogen peroxide in distilled water to block endogenous peroxidases, placed in a 10% non-immune normal goat serum (Jackson ImmunoResearch Labs), incubated with a primary antibody (rabbit anti-ChAT, 1∶1000, Abcam) and with a secondary antibody (anti-rabbit IgG peroxidase conjugate, 1∶200, KPL) in PBS. Immunoreactivity was visualised with 3,3′-diaminobenzidine in PBS containing 0.01% hydrogen peroxide. Routine immunolabelling controls were carried out by omitting the primary or secondary antibody steps, independently (for more details, see [36]). Stained and immunolabelled sections were collected onto glass, washed in PBS, dehydrated in progressive ethanol concentrations and mounted under a coverslip with a drop of DPX (Fluka). Section images were acquired using a DMR Leica microscope equipped with a High-End DP 72 Olympus digital camera and projected onto a flat computer monitor. Stereological analyses were performed using the optical-disector and the rotator procedures of the newCAST Visiopharm stereology system version 4.2.8.0.

### Stereological analysis of spinal cords

The total volumes of white and grey matter, ventral horn and lumbar spinal cord were estimated by means of the Cavalieri principle [37], [38] in the same reference sections stained with toluidine blue used for disectors. Section profile areas were estimated from the numbers of randomly-positioned test points (∼200 per compartment: white matter, grey matter and ventral horn) hitting the whole reference space and the areal equivalent of a test point. The total volumes of each compartment were obtained using the following formula V : = T. ∑A_COMP_, where T is the between-section distance (2,200 µm) and ∑A_COMP_ is the sum of the delineated profile areas of the chosen set of lumbar spinal cord compartments' sections. The volume of lumbar spinal cord was calculated by the summing the volumes of white matter and grey matter. Irrespective of groups, the mean volume shrinkage (coefficient of variation, CV, expressed as a decimal fraction of the mean) was estimated to be 2.9% (0.19) in the wild-type group, 2.1% (0.15) in the untreated G93A group and 2.2% (0.16) in the treated G93A group. No correction for global shrinkage was performed since between-group differences were not significant (p = 0.21). The optical fractionator was used for estimating the total number of toluidine blue-stained (N_TB_) and ChAT positive (N_ChAT_) neurons in spinal cord ventral horn [35], [39], [40]. Before starting the counting procedure, a z-axis distribution (calibration) was performed to determine the neuron distribution throughout section thickness and establish the disector height, which was 15 µm in the wild-type, 20 µm in the G93A untreated and treated group for both toluidine blue-stained and ChAT-positive neurons. Section thickness was measured in every field of view using the central point on the unbiased counting frame. The mean height sampling fraction (hsf) was 3/8 for toluidine blue-stained and for ChAT-positive neurons in the wild-type group and 1/2 for toluidine blue-stained and for ChAT-positive neurons in the untreated and treated G93A group. The perikaryon was defined as the counting unit. For all groups, a mean area sampling fraction (asf) of 1/7 for toluidine blue-stained and 1/6 for ChAT-positive neurons of the chosen sections was sampled using 2-D unbiased counting frames with a frame area equivalent to 10,000 µm^2^ [41]. For toluidine blue-stained neurons an average of 125 disectors were used to count 194 neurons (∑Q^−^) in the wild-type group. In the untreated G93A group, 113 disectors were applied to count 124 neurons and, in the treated G93A group, 115 disectors were applied to count 219 neurons. For ChAT-positive neurons an average of 97 disectors were used to count 187 neurons (∑Q^−^) in the wild-type group. In the untreated G93A group, 119 disectors were applied to count 107 neurons and, in the treated G93A group, 153 disectors were applied to count 234 neurons. The total number of toluidine blue-stained and ChAT-positive neurons was then estimated by multiplying the counted number of particles - neurons in this case - by the reciprocal of sampling fractions: N_TB or ChAT_: = ssf ^−1^^.^ hsf ^−1.^ asf ^−1.^ ΣQ^−^, where ssf is the mean section sampling fraction, hsf is the mean height sampling fraction, asf is the mean area sampling fraction and ΣQ^−^ is the total number of particles counted using optical disectors. The mean neuronal volume of toluidine blue-stained and ChAT-positive neurons was estimated by the planar rotator [35], [38], [42], which is a local and direct estimator of particle volume. The planar rotator method was computer assisted using the 6 half-line rotator probe available in the new CAST Visiopharm stereology system (version 4.2.8.0) and in the same reference sections used for total number estimation.

### Tissue collection and biochemical analysis

At different stages of the disease, rats were anesthetized with ketamine (90 mg/Kg) and xylazine (10 mg/Kg). Blood was collected from the right atrium of the heart into a heparin containing tube and centrifuged to separate the plasma. The plasma was stored at −80°C until EPR analysis. Blood collection was followed by perfusion of the animal with saline (0.9% sodium chloride) to eliminate the blood from the tissues before the harvesting of the spinal cords, which were stored in liquid nitrogen. The spinal cords were homogenized in extraction buffer (1∶5 g/ml) containing 50 mM Tris-HCl, pH 7.5, 150 mM NaCl, 1% Nonidet P-40, 1 mM DTPA, 1 mM sodium orthovanadate, 1 mM phenylmethyl sulfonyl fluoride, 2 µg/ml aprotinin, 2 µg/ml leupeptin and 2 µg/ml pepstatin. An Ultra Turrax T8 IKA homogenizer was employed in cycles of 15 s followed by an additional 15 s on ice. Aliquots of the homogenates were stored at −80°C until analysis. The protein concentration was determined using the Bradford method with a Bio-Rad Kit.

### Analysis of tempol and its hydroxylamine derivative by EPR

EPR spectra were recorded at room temperature on a Bruker ER 200 D-SRC that was upgraded to an EMX instrument. To determine the levels of tempol in the plasma and spinal cord homogenates of rats given 30 mM tempol as their drinking water, the animals were sacrificed between 9 AM and 10 AM, and the plasma and spinal cords were collected as described above. Aliquots of plasma and spinal cord homogenates (obtained in saline 1∶1 g/ml) were transferred to flat cells and submitted to EPR analysis before (to determine tempol levels) and after the addition of 1 mM ferricyanide (to determine tempol plus hydroxylamine levels) [29], [34]. For rats given tempol intraperitoneally (26 mg/rat), wild-type littermates were sacrificed 15, 30, 60 and 120 min after tempol administration, and the tissues were collected and analyzed as described above. The tempol concentration in the spinal cord homogenates was estimated by comparing the peak height of the first peak of the EPR spectrum with that of a standard tempol solution scanned under the same conditions [29], [34].

### Reducing capacity of spinal cord homogenates

The reducing power of the spinal cord homogenates from the wild-type and G93A rats at different disease stages and with or without tempol treatment was monitored by their ability to reduce the pre-formed radical cation of 2,2′-azinobis-(3-ethylbenzothiazoline-6-sulfonic acid) (ABTS^•+^), which was monitored at 734 nm (ε = 1.5×10^4^ M^−1^ cm^−1^) [43]. An aliquot of the spinal cord homogenates (100 µg of protein), which was obtained as described above, was added to the ABTS^•+^ solution (46.7 µM) and the absorbance was monitored for at least 100 s; the blank was the same concentration of homogenates in PBS, pH 7.4. The results are expressed as µM ABTS^•+^ reduced/min/mg of protein. The reducing powers of 15 µM tempol, hydroxylamine of tempol, Trolox and GSH were monitored as described above, and the results are expressed as µM ABTS^•+^ reduced/s.

### Analysis of carbonylated proteins in spinal cord homogenates

The levels of the carbonylated proteins in fresh spinal cord homogenates from the wild-type and G93A rats at different disease stages and with or without tempol treatment were monitored with immunoblot analysis after derivatization with 2,4-dinitrophenylhydrazine (DNPH) in trifluoroacetic acid (TFA) as previously described [44]. An aliquot of the homogenates (200 µg of protein), which was obtained as described above, was mixed with DNPH (5 mM) in 5%TFA and incubated for 15 min at room temperature. The reaction mixture was neutralized with 2 M Tris/30% glycerol, and 20 µg of protein were resolved by 15% SDS-PAGE, transferred to a PVDF membrane that was previously blocked with 5% milk-TBS (TRIS-buffered saline) and then incubated with a primary antibody (rabbit anti-DNP, 1∶2500, Sigma) and a secondary antibody (anti-rabbit IgG peroxidase conjugate, 1∶2500, KPL) in 1% milk-TBST (TBS, 0.1% tween). The immunoreactivity was detected using an enhanced chemiluminescence detection kit (Pierce, USA) and the relative quantification of carbonylated proteins was performed by densitometry (ImageJ, NIH, USA).

### Detection of a dimer-sized hSOD1 in spinal cord homogenates

A dimer-sized hSOD1 has been universally detected by SDS-PAGE western blot analysis in spinal cord extracts from ALS patients and mouse models [45]–[47]. Here, we monitored the presence of this form in spinal cord homogenates from the wild-type and G93A rats at different disease stages and with or without tempol treatment. We noticed that this dimer-sized form is detectable only in freshly prepared homogenates. Thus, a fresh aliquot of the homogenates (20 µg of protein) that was obtained as described above was incubated with Laemmli's buffer for 5 min at 100°C, resolved by 15% SDS–PAGE and transferred to a PVDF membrane that was blocked in 5% milk-TBS and then incubated with a primary antibody (sheep anti-hSOD, 1∶2000, Calbiochem) and a secondary antibody (anti-sheep IgG peroxidase conjugate, 1∶5000, Calbiochem) in 1% milk-TBST. As a standard, we used the hSOD1 dimer produced from its peroxidase activity, which was obtained and purified as previously described [48]. The immunoreactivity was detected using an enhanced chemiluminescence detection kit (Pierce, USA) and the relative quantification of dimer-sized hSod1 was performed by densitometry (ImageJ, NIH, USA).

### Detection of unfolded hSOD1 in spinal cord homogenates

Unfolded hSOD1 forms were detected with a conformational-specific antibody (USOD) targeted against hSOD1 residues 42–48, which recognizes the presence of hSOD1 with an unfolded beta barrel [49], [50]. This antibody was generously provided to us by Drs. Avijit Chakrabartty and Aaron Kerman from the University of Toronto, Canada. An aliquot of the homogenates, which were obtained as described above, was incubated at a concentration of approximately 6 mg/ml in RIPA buffer (50 mM Tris-HCl pH 7.5, 150 mM NaCl, 1% NP-40, 0.25% sodium deoxycholate, 1 mM DTPA, 0.1% SDS, 1 mM sodium orthovanadate, 1 mM phenylmethyl sulfonyl fluoride, 2 µg/ml aprotinin 2 µg/ml leupeptin and 2 µg/ml pepstatin) for 15 min at room temperature with mild shaking. After the incubation, the sample was centrifuged at 15000 *g* for 15 min at 4°C, the supernatant was collected and the protein concentration was determined with the Bradford method using a Bio-Rad Kit. Then, 2 mg of protein was incubated with 2 µg of affinity-purified USOD antibody and 50 µl of 50% Protein A-Agarose (Upstate) in RIPA buffer (750 µl final volume) overnight at 4°C. After the incubation, the sample was centrifuged at 5000 *g* for 5 min. The pellet was washed 3 times with cold RIPA buffer, eluted with Laemmli's buffer, incubated for 5 min at 100°C, resolved by 15% SDS–PAGE and transferred to a PVDF membrane, which was blocked in 5% milk-TBS and then incubated with a primary antibody (sheep anti-hSOD, 1∶2000, Calbiochem) and a secondary antibody (anti-sheep IgG peroxidase conjugate, 1∶3000, Calbiochem) in 1% milk-TBST. A separate blot loaded with the supernatants from each sample (20 µg of protein) was performed as a loading control. The immunoreactivity was detected using an enhanced chemiluminescence detection kit (Pierce, USA) and the relative quantification was performed by densitometry (ImageJ, NIH, USA).

### Statistical analysis

Kaplan–Meier survival curves were generated using GraphPad Prism and compared using the long-rank test. A P-value of less than 0.05 was considered statistically significant. Body weights, motor scores and the biochemical data are expressed as the mean ± standard error. The statistical significance was calculated using one-way ANOVA. The statistical significance for body weight and motor score analysis was calculated using two-way ANOVA followed by Bonferroni post-test. The precision of a stereological estimate was expressed as a coefficient of error (CE) calculated as described by Gundersen *et al*. [37]. The stereological data were expressed as group mean (observed coefficient of variation, CV_obs_) where CV_obs_ represents standard deviation/mean. Group differences were assessed by the non-parametric Kruskal-Wallis test and by one-way analysis of variance (ANOVA) using Minitab version 16 (2010) and SAS version 9.2 statistical software. When significant between-group differences (p<0.05) were noted, Tukey's test for multiple comparisons was applied.

## Results

### Effects of oral administration of tempol on ALS progression in G93A rats

**Figure 1:**
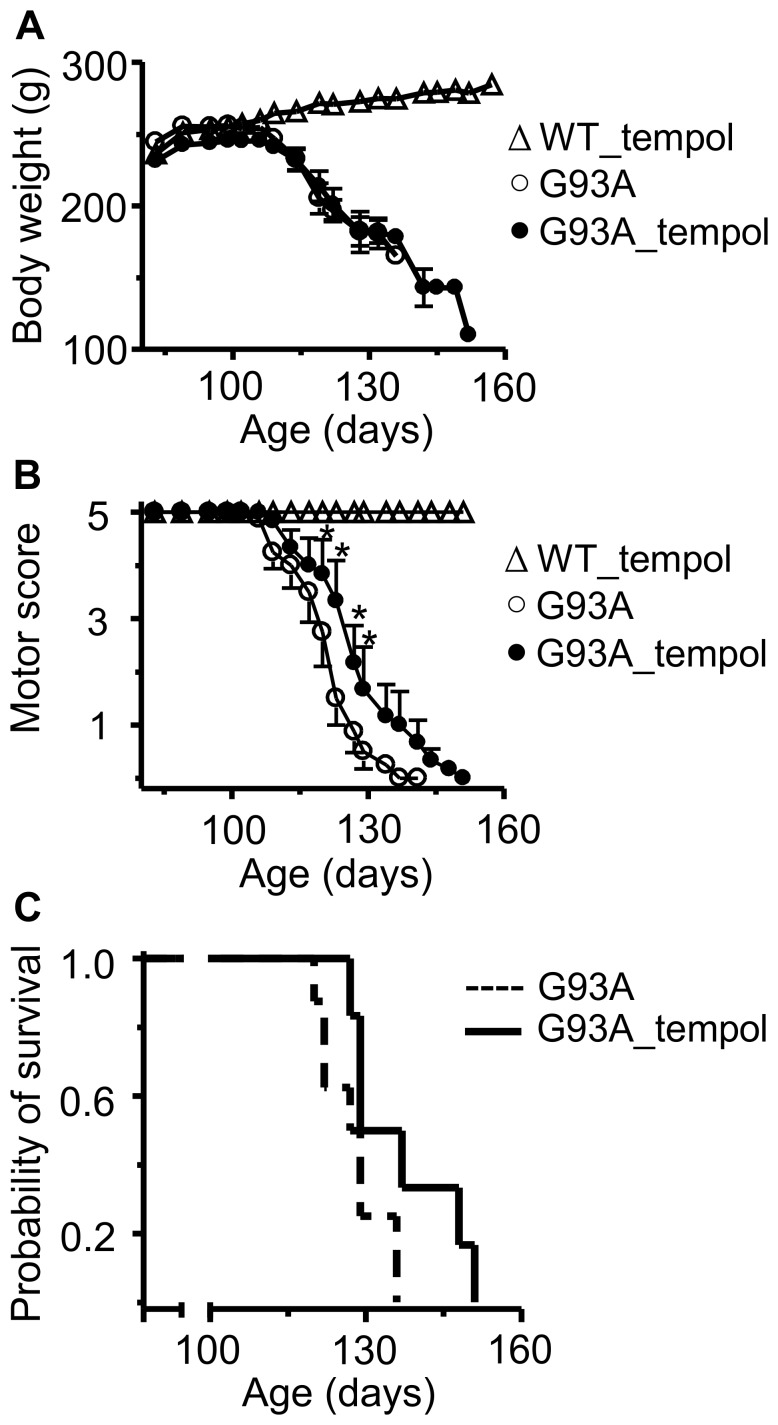
Effects of oral tempol on ALS progression in G93A rats. A) The body weight loss in G93A rats untreated (n = 11) and treated (n = 9) with tempol; wild-type rats treated with tempol (n = 11) exhibited similar behavior to those not treated (not shown). Female rats received tempol (30 mM) in their drinking water starting at 7 weeks of age. B) The motor scores of the same animals were determined as described in the Materials and Methods; the values shown correspond to the mean ± standard error; *p<0.05 (two-way ANOVA with Bonferroni post-test). C) The survival analysis of untreated and treated G93A rats. The results are shown as a Kaplan-Meier plot and are significantly different at p<0.05 (log-rank test). The end points of the non-treated and treated rats were 127.6±2.2 and 136.8±4.3 days, respectively.

Female rats expressing the mutant hSOD1^G93A^ (7 weeks old) were randomly divided in tempol-treated and untreated littermates, and the wild-type littermates were used as control animals. The first experimental group of animals were given a tempol solution (5 mM) as their drinking water *ad libitum* (n = 10) [29], [34] and the untreated animals (n = 11) received water; the wild-type littermates received water (n = 10) or tempol (n = 10). Disease progression was evaluated by monitoring the body weight and the motor score of the rats according to the method described in the Materials and Methods [31]. In contrast to the continuous increase of the body weight of wild-type rats, those expressing hSOD1^G93A^ reached a maximum body weight around at 97.8±2.7 days of age that was then followed by a continuous decline, which is a characteristic of the ALS disease [22], [31]. Treatment with 5 mM tempol in the drinking water did not affect the weight loss, the motor performance or the survival of the animals (data not shown). Considering the low toxicity of tempol, we increased its dosage for the second experimental group of rats; they were given a solution of 30 mM tempol (n = 9) as their drinking water *ad libitum* and the untreated animals (n = 11) received water. The wild-type littermates received water (n = 10) or tempol (n = 12). The animals consumed similar volumes of water or the tempol solution (approximately 30 ml/day), which resulted in approximately 155 mg of tempol/day/rat. At this high dosage, the tempol treatment slightly improved the motor score and extended the rat´s survival by 9 days (the end points for untreated and treated animals were 127.6±2.2 and 136.8±4.3, respectively) (Figure 1). The characteristic weight loss accompaining disease evolution in G93A rats was not affected by tempol treatment (Figure 1). This could result from a slimming effect of the nitroxide. Indeed, long-term administration of high doses of tempol to mice has been shown to obviate weight gain from food supplementation without affecting food intake, metabolism or the level of activity of the animals [30], [51], [52]. However, under our experimental conditions, wild-type rats, treated or untreated with tempol, displayed similar normal behavior with regard to weight gain and motor performance up to 6 months of age (Figure 1, and data not shown). These results argue against a slimming effect of tempol although we cannot exclude it.

**Figure 2:**
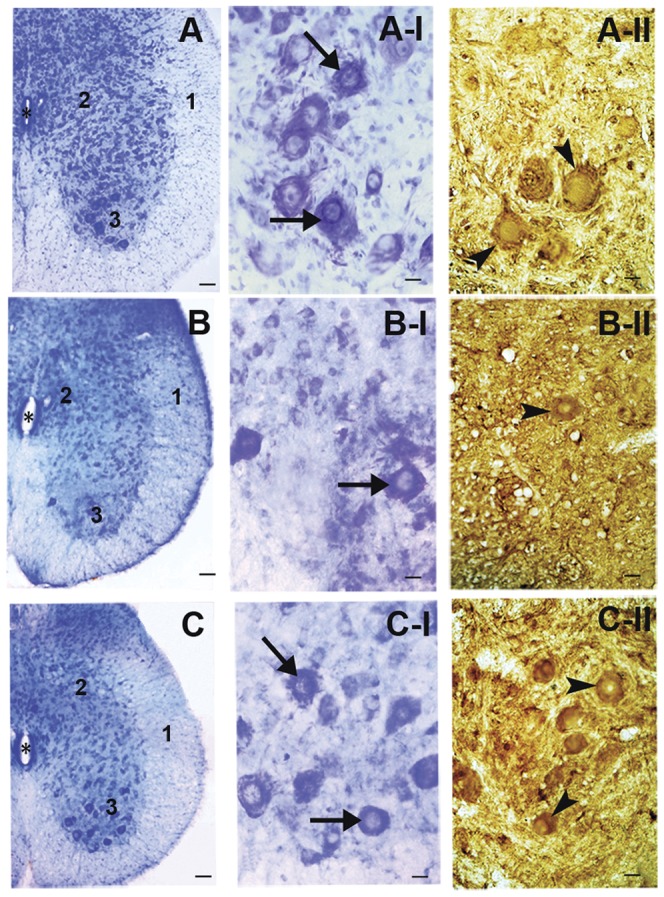
Effects of oral tempol on neuron degeneration in the lumbar spinal cord from G93A rats. Representative light-microscopic images of toluidine-blue stained (A, A-I, B, B-I, C and C-I) and ChAT immunolabeled (A-II, B-II and C-II) sections of the lumbar spinal cord from wild-type and G93A rats both untreated and treated with oral tempol. Female rats received tempol (30 mM) in their drinking water starting at 7 weeks of age and were sacrificed at the time of middle symptomatic phase of G93A rats (115–120 days). A) wild-type rats; B) untreated G93A rats; and C) treated G93A rats. White matter (1), grey matter (2), ventral horn (3) and the central canal (*) are labeled as specified. Also, note the toluidine blue-stained (arrows in A-I, B-I and C-I) and immunolabeled ChAT-positive (arrowheads in A-II, B-II and C-II) neurons. Scale bars: 100 µm (A, B and C) and 20 µm (A-I, B-I, C-I, A-II, B-II and C-II). Stereological methods were conducted in 15 rats, 5 animals per each group: wild-type, untreated G93A and treated G93A. Ten 40 µm-thick systematic, uniform and random sections were analyzed per animal, summing up 50 sections per group and 150 sections in the 3 studied groups.

The modest protective effect of oral administration of tempol on the behavior of G93A rats led us to examine if this treatment delayed neurodegeneration. A parallel group of G93A rats untreated (n = 5) and treated (n = 5) with 30 mM tempol in the drinking water as above and wild-type littermates (n = 5) were sacrificed at the time of middle symptomatic phase of G93A rats (115–120 days) and their spinal cords were histologically and stereologically analyzed (see, Materials and Methods). There were no significant differences in the volume of the lumbar spinal cord and of its anatomical regions in the three groups of animals (Table S1). However, as expected, the untreated G93A group presented neuron losses and atrophy as compared with the wild-type group (Figure 2A, A-I, A-II and 2B, B-I, B-II; Table 1). Treatment with tempol significantly protected against neuron losses but did not prevent atrophy (Figure 2C, C-I, C-II; Table 1). These results suggest that tempol may attenuate the processes triggering neuron damage without halting the on-going neurodegeneration.

**Table 1:** Stereological analysis of the effects of oral tempol in spinal cords.

Stereological parameters	Wild-type	Untreated G93A	Treated G93A
**TB neurons**			
Total number*	202,569 (0.08)^a^	96,054 (0.04)^b^	169,300 (0.19)^a^
Mean volume (µm^3^)**	23,645 (0.12)^a^	14,129 (0.06)^b^	16,868 (0.10)^b^
**ChAT-positive neurons**			
Total number***	167,314 (0.24)^a^	70,763 (0.05)^b^	154,930 (0.24)^a^
Mean volume (µm^3^)****	32,608 (0.16)^a^	16,377 (0.13)^b^	18,807 (0.02)^b^

Stereological estimates for total number and mean volumes (µm^3^) of toluidine-blue stained neurons (TB neurons) and ChAT-positive neurons in lumbar spinal cord of G93A rats untreated and treated with oral tempol. Rats received tempol (30 mM) in their drinking water starting at 7 weeks of age and were sacrificed at the time of middle symptomatic phase of G93A rats (115–120 days) Values are group means (CVs).

For toluidine blue-stained neurons, the precision of estimated number (expressed as CE(N_TB_)) was 0.04 for the wild-type group, 0.06 for the treated G93A group and 0.02 for the untreated G93A group. For ChAT-positive neurons the precision of estimated number (expressed as CE(N_ChAT_)) was 0.07 for the wild-type group, 0.06 for the treated G93A group and 0.02 for the untreated G93A group. Means that not share a letter are significantly different (*p = 0.002; **p = 0.003; ***p = 0.02; ****p = 0.002). Stereological methods were conducted in 15 rats, 5 animals per each group: wild-type, untreated G93A and treated G93A. Ten 40 µm-thick systematic, uniform and random sections were analyzed per animal, summing up 50 sections per group and 150 sections in the three studied groups.

**Figure 3:**
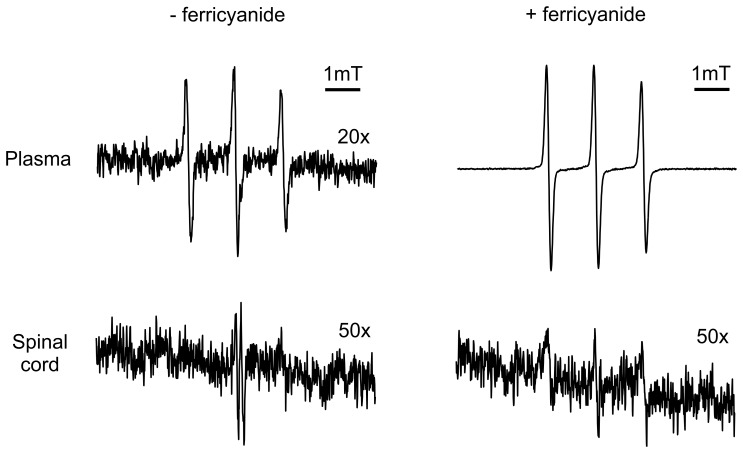
Representative EPR spectra of tissues of G93A rats treated with oral tempol. Female rats received tempol (30 mM) in their drinking water starting at 7 weeks of age and were sacrificed at the symptomatic phase in the morning. The spectrum of each sample was scanned before and after addition of 1 mM ferricyanide, as specified in the figure. The shown spectra are representative of the spectra obtained from homogenates of 3 different symptomatic G93A rats treated with oral tempol. Instrumental conditions: microwave power, 10 mW; modulation amplitude, 0.1 mT; time constant, 163 ms; scan rate, 0.060 mT/s.

Both functional and analytical studies have demonstrated that tempol penetrates the blood-brain barrier (reviewed in [28]), which was confirmed by EPR analysis of the plasma and spinal cord homogenates of rats treated with 30 mM tempol in their drinking water (Figure 3). Most of the tempol was present in its reduced form, the corresponding hydroxylamine, which is typical of biological fluids and tissues. Indeed, the characteristic EPR signal of tempol became noticeable upon the addition of ferricyanide to the plasma and spinal cord homogenates [29], [34] (Figure 3). The estimated concentration of tempol plus hydroxylamine in the plasma and spinal cord homogenates of the treated, symptomatic rats was in the low µM to tenth of µM range (1–3 and c.a. 0.1 µM, respectively). Collectively, these results show that the levels of tempol plus hydroxylamine attained in the central nervous system slightly ameliorated the course of ALS disease in the G93A rat model (Figures 1, 2, 3; Table 1).

### Antioxidant action of tempol in spinal cords from G93A rats

**Figure 4:**
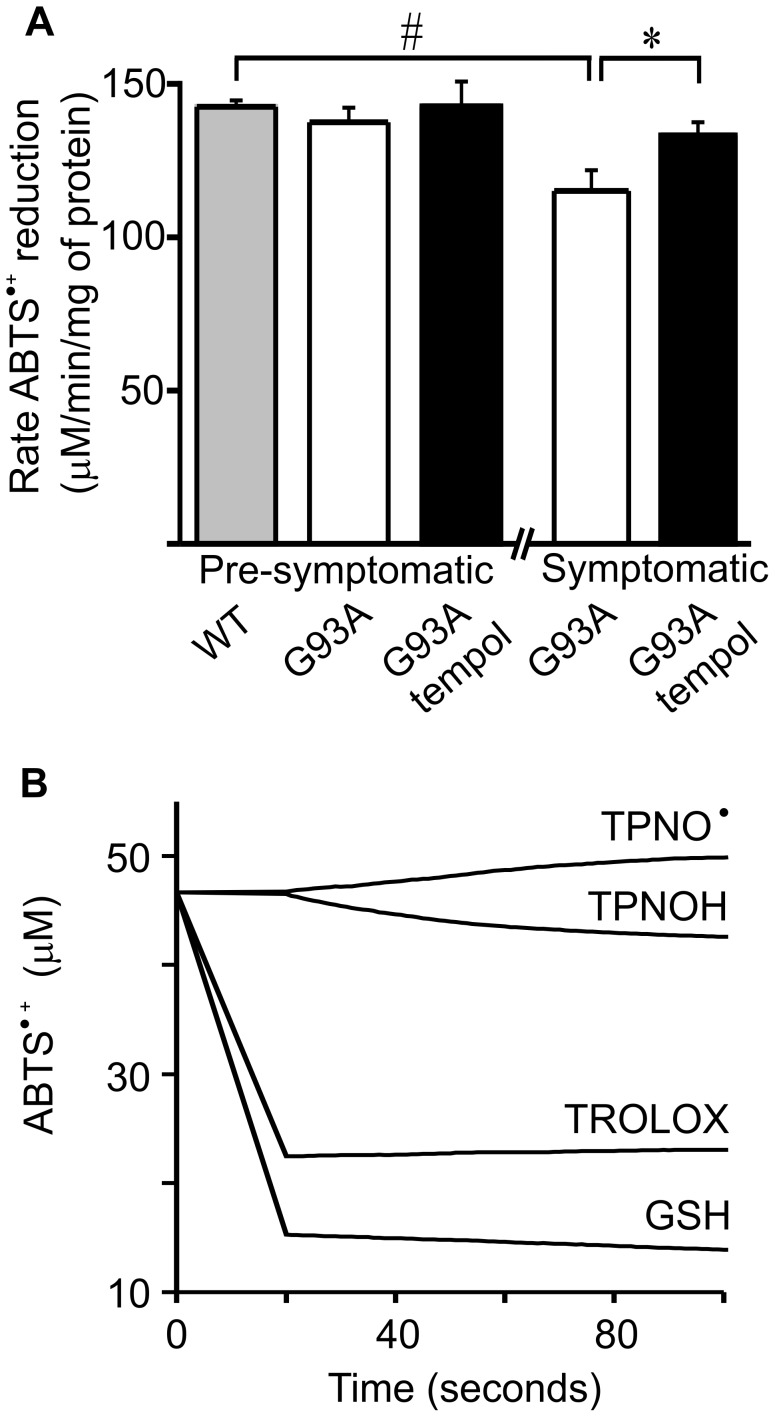
Effect of oral tempol on the reducing power in spinal cord homogenates of G93A rats. Relative quantification of the reducing power of spinal cord homogenates of female G93A rats, both treated and untreated with oral tempol. A) The reducing capability to reduce ABTS^•+^ of homogenates of G93A rats at different disease stages compared with that of wild-type rats; the values shown correspond to the mean ± standard error of the values obtained from homogenates of different animals at the pre-symptomatic (wild-type (n = 5), untreated G93A (n = 4) and treated G93A (n = 4)) and symptomatic phase (untreated G93A (n = 6) and treated G93A (n = 6)); ^#^p<0.01 and *p<0.05 (one-way ANOVA). Female rats received tempol (30 mM) in their drinking water starting at 7 weeks of age. B) A comparison of the ability of equal concentrations (15 µM) of tempol (TPNO^•^), tempol-derived hydroxylamine (TPNOH), Trolox and GSH to reduce the ABTS^•+^ in phosphate buffer, pH 7.4, 25°C.

To gain insight into the mechanisms by which tempol affects ALS disease in rats, we examined whether it displayed antioxidant actions in the spinal cords of the animals. It should be noted that the rapid reduction of tempol in vivo (Figure 3) [28] shows that, in the short run, tempol consumes endogenous reducing agents, such as ascorbic acid [53], [54] and NADPH [55]. However, in the long run, tempol usually displays antioxidant actions as has been demonstrated in several animal models of diseases [25]–[30]. In the G93A rat model, we first compared the reducing power of spinal cord homogenates of tempol-treated and untreated rats towards ABTS^•+^ [43] in different phases of the disease. The homogenates from wild-type rats exhibited the highest capacity to reduce ABTS^•+^, followed by homogenates from pre-symptomatic and symptomatic G93A rats, respectively (Figure 4A). The differences were small, but they indicated that the disease evolution consumes the reducing capacity of spinal cord tissues, which were protected by treatment with tempol in a statistically significant manner (Figure 4A). Control experiments showed that tempol does not reduce ABTS^•+^ at measurable rates in vitro; however, the hydroxylamine of tempol does reduce ABTS^•+^ with low efficiency as compared with classical antioxidants (Figure 4B). Considering that the instantaneous concentration of hydroxylamine plus tempol in spinal cord homogenates is c.a 0.1 µM (Figure 3), it cannot be directly responsible for recovering the reducing capability of the homogenates of treated rats. Most likely, tempol is attenuating the oxidative processes that lead to the consumption of the endogenous reductants in spinal cord tissues.

**Figure 5:**
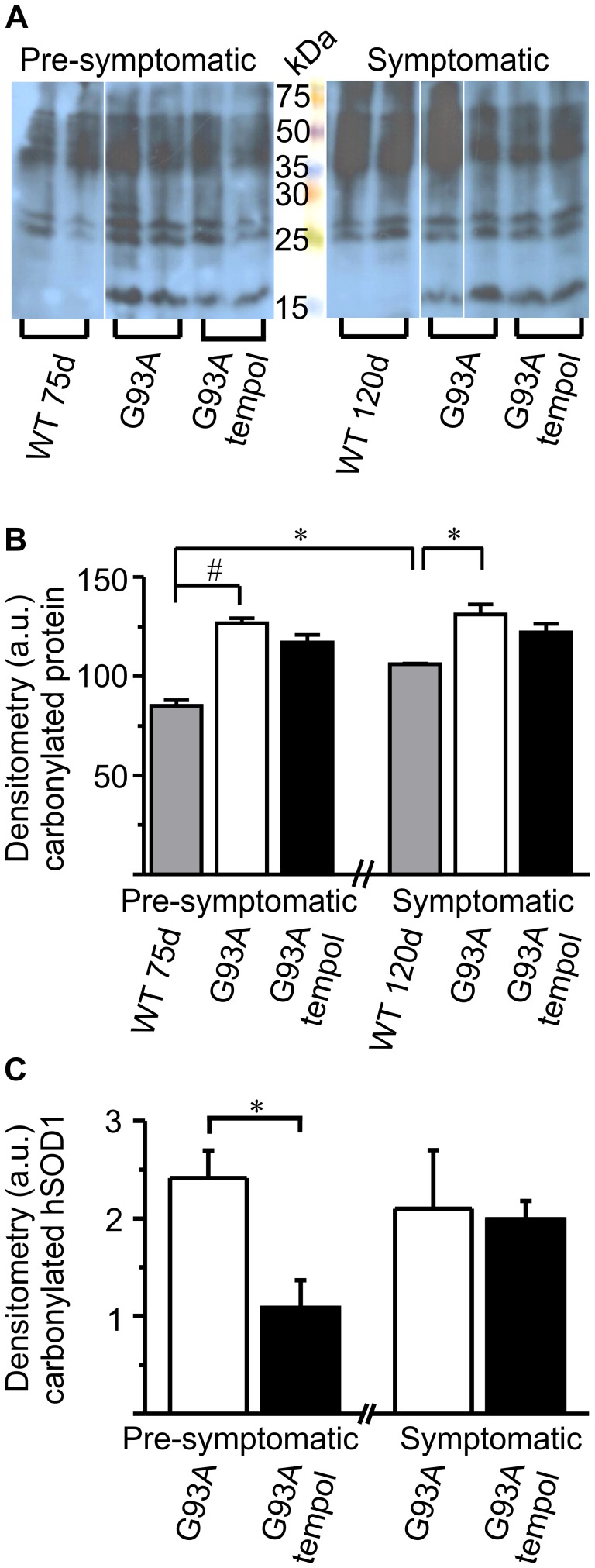
Effect of oral tempol on carbonylated protein levels in spinal cord homogenates of G93A rats. Relative quantification of the levels of carbonylated proteins in spinal cord homogenates of G93A rats both treated and untreated with oral tempol. A) Representative western blots of carbonylated proteins in spinal cord homogenates at different stages of the disease compared with that of wild-type rats. Female rats received tempol (30 mM) in their drinking water starting at 7 weeks of age. The homogenates from 16 rats at the pre-symptomatic (wild-type (n = 2), untreated G93A (n = 3) and treated G93A (n = 3) and symptomatic phase (wild-type (n = 2), untreated G93A (n = 3) and treated G93A (n = 3)) were obtained and analyzed in parallel in 2 gels as described in the Materials and Methods. Representative lanes of the western blots were arranged in A) to facilitate visual comparison. B) The relative quantification of the carbonylated proteins by densitometry of the corresponding whole lanes in the western blots. C) The relative quantification of the carbonylated hSOD1^G93A^ by densitometry of the bands at approximately 15 kDa in A). The values shown correspond to the mean ± standard error of the results obtained from homogenates of the 16 different rats specified above; ^#^p<0.01 and *p<0.05 (one-way ANOVA).

Another marker of bulky oxidative damage that was monitored in the spinal cord homogenates was the level of carbonylated proteins. In agreement with previous results reported in ALS mouse models [15], [56], [57], spinal cord homogenates from G93A rats exhibited higher total levels of carbonylated proteins than those of wild-type rats but the levels did not significantly increase from the pre-symptomatic to the symptomatic phase (Figure 5A and 5B). This may be due to saturation of the possible steady-state levels of carbonylated proteins in the spinal cords of G93A rats because heavily oxidized proteins are usually mobilized to degradation [58]. Wild-type rats showed the expected increase of the total level of protein carbonylation with age [59] (Figure 5A and 5B). Treatment with tempol tended to decrease the total levels of carbonylated proteins in spinal cords homogenates of G93A rats but the differences were not statistically significant (Figure 5A and 5B). However, tempol significantly decreased the levels of carbonylated hSOD1 in the pre-symptomatic but not in the symptomatic phase of the disease (Figure 5A and 5C). Human SOD1 corresponds to the pronounced band at approximately 15 kDa in Figure 5A, which is absent in the blot of homogenates from wild-type rats. These results further indicate that oxidative processes leading to saturating levels of protein carbonylation occurs early in the disease. Accordingly, it has been recently reported that lymphocytes from a subset of sporadic ALS patients with bulbar onset presented high levels of carbonylated wild-type hSOD1, the levels of which were not increased by treating the cells with the oxidant hydrogen peroxide [60].

Monitoring parameters of bulky oxidative damage in the spinal cord homogenates of G93A rats showed that these parameters increase moderately in the course of the disease as compared with the evolution of the symptoms (Figures 1, 2, 4 and 5). Nevertheless, the monitoring of these parameters showed that treatment with tempol did not consume the pool of reductants in the spinal cords [53]–[55] but, rather, protect it significantly (Figure 4). Also, the results showed that treatment with tempol tended to attenuate the generalized oxidation of proteins to carbonylated proteins and to decrease the carbonylation of hSOD^G93A^ in a statistically significant manner (Figure 5). Therefore, the results are in line with the view that redox processes are compartmentalized [61], [62], and damage to specific targets may be more relevant to trigger toxic mechanisms. Thus, we further examined the effects of tempol on disease-triggered modifications of the specific target, hSod1^G93A^, whose over-expression in the rats is the causative agent of ALS symptoms.

### Tempol inhibits the level of non-native hSod1 forms present in spinal cords from G93A rats

**Figure 6:**
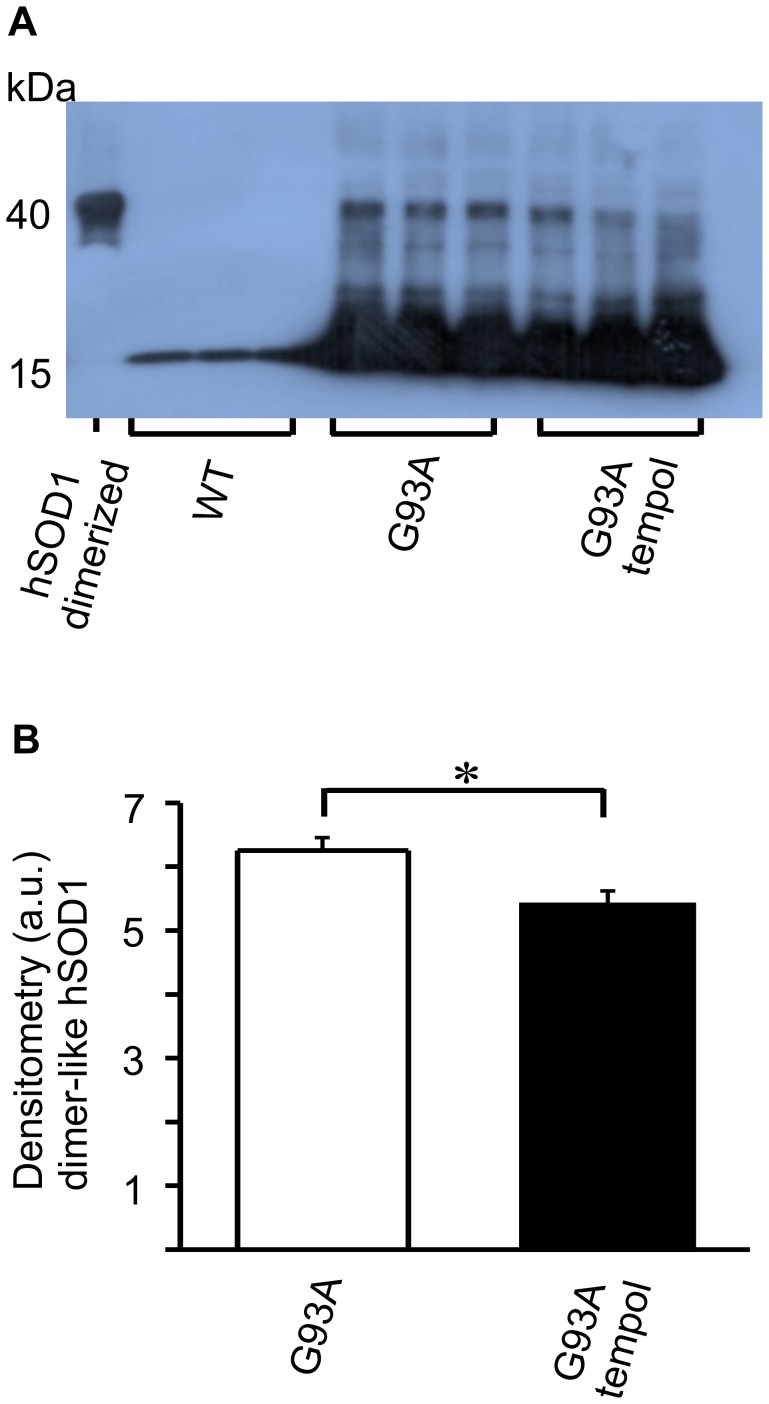
Effect of oral tempol on dimer-sized SOD1 levels in spinal cord homogenates of G93A rats. Relative quantification of the levels of dimer-sized SOD1 species in spinal cord homogenates of G93A rats both treated and untreated with oral tempol. A) Western blot analysis of spinal cord homogenates obtained at the symptomatic phase compared with that of wild-type rats. The blots were visualized with an anti-SOD1 antibody; the dimer-sized SOD1 species that was used as standard was obtained through the enzymés peroxidase activity as previously described (48). Female rats received tempol (30 mM) in their drinking water starting at 7 weeks of age. B) The relative quantification of the dimer-sized SOD1 species by densitometry of the respective bands shown in A. The values shown correspond to the mean ± standard error of the results obtained with homogenates from 3 different rats from each group: wild-type, untreated G93A and treated G93A; *p<0.05 (one-way ANOVA).

Despite many advances in recent years, there is no consensus with regard to the nature of the modification(s) responsible for the toxicity of ALS-SOD1 mutants [2]–[9]. In addition to carbonylated hSOD1 [45], [56], [57], [60], other modified hSOD1 forms have been detected during the course of ALS disease and have been considered to be potentially toxic [45]–[50]. One of these non-native hSOD1 species is a thiol-resistant and dimer-sized species, which is universally detected by SDS-PAGE western blots of spinal cord extracts from ALS patients and mouse models [45]–[48]. Here, we show that this dimer-sized hSOD1 species is also present in the spinal cord homogenates of ALS rats in the symptomatic phase, and that its level is significantly decreased upon treatment with tempol (Figure 6). In agreement with the literature [45], [47], [56], the dimer-sized hSOD1 species was barely detectable in spinal cord homogenates from rats in the pre-symptomatic phase. We also observed that the dimer-sized hSOD1 species is more easily detectable in fresh homogenates, probably because it aggregates with time in frozen homogenates (data not shown).

**Figure 7:**
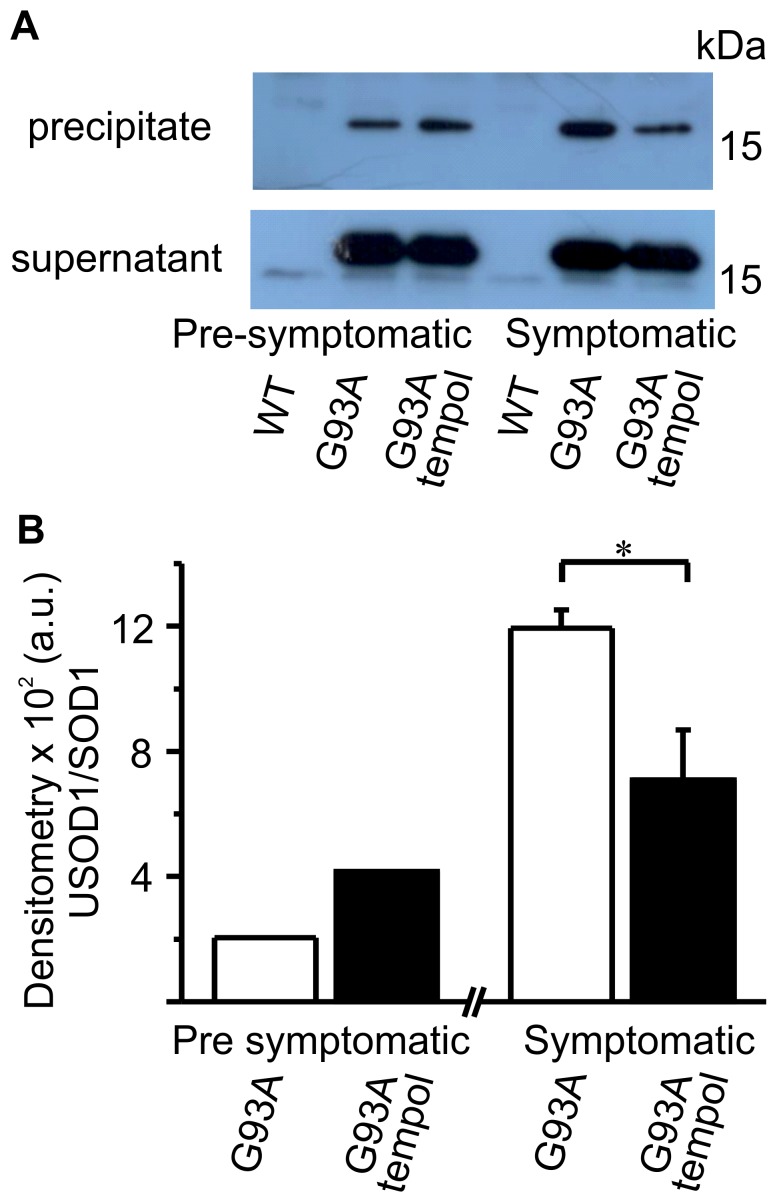
Effects of oral tempol on unfolded SOD1 forms in spinal cord homogenates of G93A rats. Relative quantification of the levels of unfolded SOD1 forms in spinal cord homogenates of G93A rats both treated or untreated with oral tempol. Female rats received tempol (30 mM) in their drinking water starting at 7 weeks of age. A) Representative western blots of the supernatant and precipitate of the spinal cord homogenates obtained at different stages of the disease that were immunoprecipitated with the USOD antibody as described in the Materials and Methods. All the blots were visualized with an anti-SOD1 antibody. B) The relative quantification of unfolded hSOD1 forms (precipitate) and monomer SOD1 (supernatant) by densitometry of the respective bands obtained in western blots of spinal cord homogenates. The values shown correspond to the mean of the results obtained with homogenates from 2 different rats from each group (pre-symptomatic phase) and mean ± standard error of the results obtained with 3 different rats from each group (symptomatic phase); *p<0.05 (one-way ANOVA).

Other non-native hSOD1 forms that are receiving increased attention in the literature are the unfolded/misfolded forms. Therefore, different conformational-specific antibodies are being developed and tested in spinal cord samples from ALS patients and animal models [49], [50], [63], [64]. The conformational-specific antibody (USOD) that is targeted against hSOD1 residues 42–48 and recognizes hSOD1 with an unfolded beta barrel [49], [50] was generously provided to us and used to test our samples. The immunoprecipitation reaction using the USOD antibody and spinal cord homogenates from G93A rats was performed as described in the Materials and Methods, and the western blots of the immunoprecipitates and supernatants are shown in Figure 7. A small fraction of total hSOD1 appears to be misfolded in the pre-symptomatic phase, but this fraction increases in the symptomatic phase. Treatment with tempol attenuates the increase in misfolded hSOD1 forms in the spinal cord homogenates (Figure 7). Collectively, these results indicate that the treatment of G93A rats with tempol decreased the levels of non-native hSOD1 forms present in spinal cord homogenates in the symptomatic phase (Figures 5C, 6 and 7).

### Effects of i.p. administration of tempol on ALS progression in G93A rats that unexpectedly diverted to decelerated disease progression

**Figure 8:**
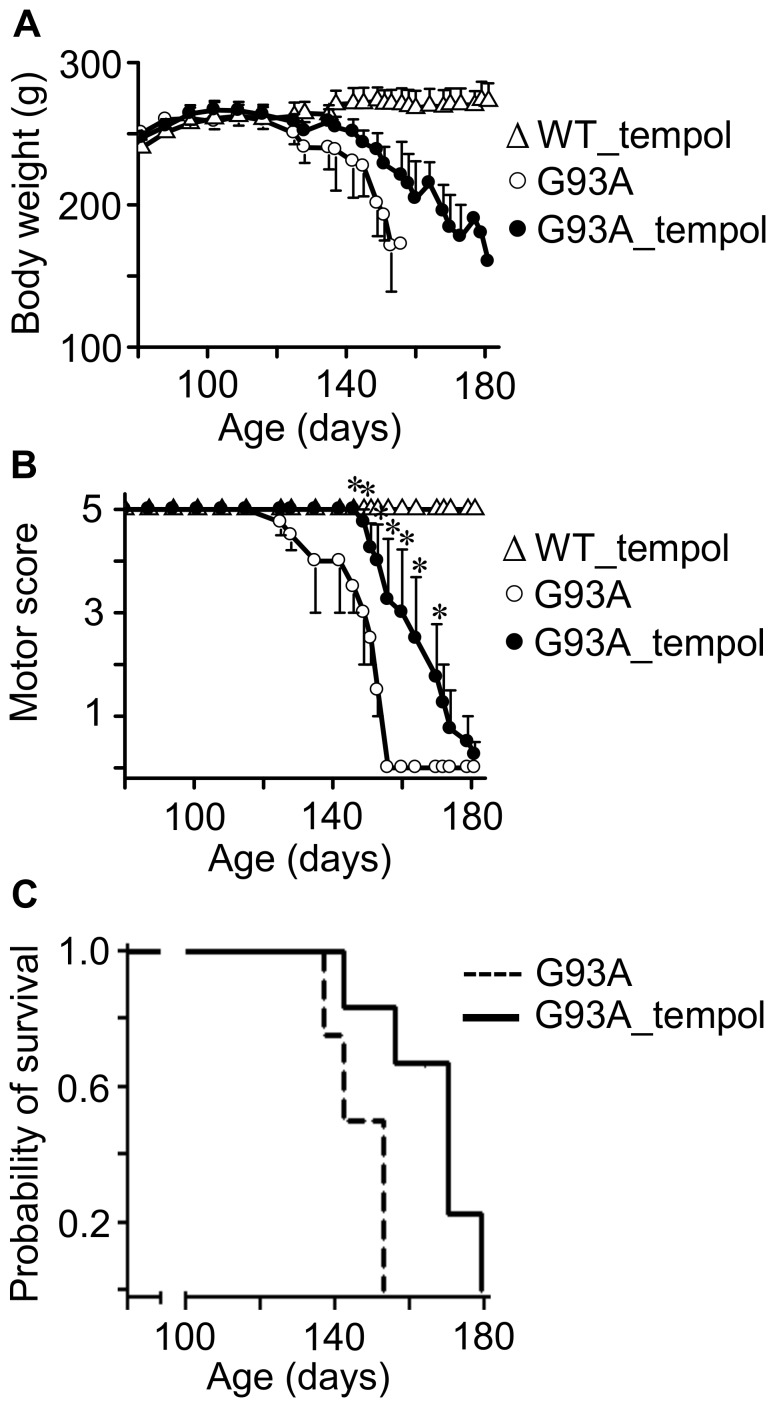
Effects of i.p. tempol on ALS progression in G93A rats. A) The body weight loss in G93A rats untreated (n = 8) and treated (n = 15) with tempol; wild-type rats treated with tempol (n = 8) exhibited similar behavior to those not treated (not shown). Female rats received tempol (26 mg/rat) each Monday, Wednesday and Friday starting at 7 weeks of age. B) The motor scores of the same animals determined as described in the Materials and Methods; the values shown correspond to the mean ± standard error; *p<0.05 (two-way ANOVA with Bonferroni post-test). C) The survival analysis of untreated and treated G93A rats. The results are shown as a Kaplan-Meier plot and are significantly different at p<0.05 (log-rank test). The end points for non-treated and treated animals were 146.3±4.0 and 163.4±6.5 days, respectively.

In view of the modest but consistent protective effects of administering a tempol solution (30 mM) as the drinking water on symptoms, neuron loss and protein modification of G93A rats (Figures 1, 2, 3, 4, 5, 6, 7), we decided to examine whether i.p. administration could be more effective. Drug dosage is more reliable in an i.p. administration because it does not depend on the volume of water consumed, and because decomposition in the stomach is avoided. On the other hand, i.p. administration is labor- and time-consuming. The i.p. dosage chosen was based on the literature [28], [29], [65] and on our preliminary observation that a few rats receiving 52 mg of tempol i.p. exhibited mild convulsions. Thus, the third experimental group of animals received tempol (26 mg in 0.5 ml saline/animal) intraperitoneally each Monday, Wednesday and Friday (n = 15) morning while the untreated animals received saline intraperitoneally (n = 8). With this i.p. treatment, tempol had a particularly significant effect in extending the survival of the G93A rats by 17 days (the end point for untreated and treated animals was 146.3±4.0 and 163.4±6.5, respectively) (Figure 8). The protective effects on motor symptoms and the absence of statistically significant effects on weight loss were similar to those provided by the oral treatment (compare Figures 1 and 8). However, a confounding factor arose when the rats that constituted the third experimental group unexpectedly diverted to a decelerated disease progression. Disease onset for the untreated rats monitored by peak weight was similar for the third (100.3±5.3 days) and the first and second group of rats (97.8±2.7 days). However, the survival of the untreated rats increased from 127.6±2.2 days in the first two groups (Figure 1) to 146.3±4.0 days in the third group (Figure 8). Similar phenotypic drifts have been reported by other investigators to the rat provider, Taconic, which is developing a new ALS rat colony.

The higher protective effect of i.p. tempol (26 mg/rat/day on every Monday, Wednesday and Friday) (Figure 8) when compared with tempol in the drinking water (approximately 155 mg/rat/day) (Figure 1) may result from a higher tempol bioavailability. The instantaneous levels of tempol plus hydroxylamine in biological fluids and tissues of the rats provided by i.p. administration are much higher than those of oral administration. Rats that received tempol in the drinking water exhibited levels of tempol plus hydroxylamine in the low µM range in plasma (Figure 3). In contrast, rats that received tempol i.p. presented levels of tempol plus hydroxylamine of 700, 600, 200 and 40 µM at 15, 30, 60 and 120 min after the injection, respectively (see, for instance, Figure S1). Similarly, the levels of tempol plus hydroxylamine in the spinal cord homogenates of rats given oral tempol were extremely low (Figure 3); whereas the levels from the rats given tempol i.p. that were sacrificed 15 and 60 min after the injection were 70 and 40 µM, respectively (Figure S1). Alternatively, the higher protective effect of a lower i.p. dosage compared with that consumed in the drinking water on the survival of G93A rats may be consequence of the decelerated disease progression of the rats that received tempol i.p., which would provide a longer time window for the action of tempol. Other reports in the literature show that different types of interventions are more effective in animal models that express low copy numbers of mutant hSOD1, which also exhibit decelerated disease development [66], [67].

## Discussion

Our results show that the administration of a high dose of tempol in the drinking water (approximately 155 mg/day/rat) of female G93A rats starting at the age of 7 weeks had marginal effects on disease onset but resulted in a slightly decelerated disease progression and extended the survival of the animals by 9 days (Figure 1). This improved animal behavior was associated with protective effects in spinal cord tissues as evidenced by the sustained number of neuronal cells (Figure 2, Table 1) and the sustained levels of reducing agents (Figure 4). In addition, carbonylated hSOD1 (Figure 5C) and non-native hSOD1 forms (Figures 6 and 7), which are considered to be crucial to neurotoxic mechanisms [2]–[9], [49], [50], [60], [63], [64], were present in lower levels in spinal cord homogenates from rats treated with tempol. The modest but consistent effects of tempol on disease evolution (Figure 1) and on markers of cellular (Figure 2, Table 1) and molecular damage (Figures 5C, 6 and 7) prompted us to try to increase tempol bioavailability by using an i.p. administration (Figures 8 and S1). In the latter case, a much lower dose of tempol (26/mg/rat/day three times a week) extended the survival of the animals by 17 days (Figure 8). However, a confounding factor arouse due to the rats in this experimental group unexpected diverting to a decelerated disease progression. Consequently, the higher protective effect of a lower i.p. dose, as compared with that consumed in the drinking water, may be due to higher tempol bioavailability (Figure S1), to decelerated disease development or both. The phenotypic variation of the rat colony available to us made further experiments with it unattractive at this point.

In contrast with mouse models, the current rat models over-expressing hSOD1 mutants show variability with respect to the site of disease onset and individual life span independently from the levels of mutant hSOD1 [22]. This variability is disadvantageous for drug-testing trials, but it recapitulates the variable phenotype of the human disease. Another advantage of ALS rat models is the larger size of the animals, which provides more tissues for the analysis of biomarkers. In our investigation, it was possible to show that the modest effect of tempol in ameliorating ALS progression in the employed rat model (Figures 1 and 8) was associated with cellular (Figure 2, Table 1) and molecular protection (Figures 4, 5, 6, 7).

The fact that treatment with tempol partially preserved spinal cord tissues against oxidative processes is consistent with the view that oxidative damage contributes to the neurodegenerative process in ALS [11]–[17], [60]. Indeed, tempol reacts with diverse biological oxidants and reductants while being recycled through the oxammonium cation- (TPNO^+^) and hydroxylamine-derivative (TPNOH), respectively. This attribute permits an effective antioxidant action in vivo because tempol can deactivate several reactive species before being consumed/metabolized [25]–[30]. In addition, the rapid reduction of tempol to the corresponding hydroxylamine in vivo (Figures 3 and S1) creates, in the short run, a redox imbalance. This imbalance, in turn, may trigger an adaptive response leading to increased antioxidant defenses in the long run [28]. An overall antioxidant action of tempol in the ALS rat model was evidenced by its protective effects on the levels of reductants (Figure 4), oxidized/carbonylated hSOD1 (Figure 5C) and non-native hSOD1 forms (Figures 6 and 7) in spinal cord tissues. Although these non-native forms may result from inherent properties of the hSOD1^G93A^ mutant structure under physiological conditions [5]–[8], they also result from oxidative attack on hSOD1 [48], [49], [56], [60], [63], [68]. Relevantly, it has been reported that over-oxidized/carbonylated wild-type hSOD1 is present in lymphocytes of sporadic ALS patients [60]. This finding identifies a possible common hSOD1-dependent toxic mechanism between familial ALS and a subset of sporadic ALS [60]. However, to establish all the possible connections between oxidized and non-native hSOD1 forms, it is necessary to structurally characterize these forms. Up to this point, modified hSOD1 forms have been usually identified by immuno-based techniques that provide limited structural information.

In conclusion, our study showed that the administration of the cyclic nitroxide tempol before disease onset (7 weeks of age) to females of an ALS rat model moderately extended the survival of the animals while protecting their cellular and molecular structures against damage. Therefore, our investigation provides proof for the hypothesis that multifunctional cyclic nitroxide antioxidants are alternatives that are worth further testing in animal models of ALS. In this case, pre-clinical drug tests following recently established guidelines [22] in specialized testing facilities [23] should be pursued.

## Supporting Information

Figure S1 **Representative EPR spectra of tissues of G93A rats treated with i.p. tempol.** The animals received tempol i.p. (26 mg/rat) and were euthanized at the different times specified in the figure. The spectrum of each sample was scanned before and after the addition of 1 mM ferricyanide. The shown spectra are representative of the spectra obtained from homogenates of 3 different G93A rats (pre-symptomatic phase) treated with i.p. tempol. Instrumental conditions: microwave power, 10 mW; modulation amplitude, 0.1 mT; time constant, 163 ms; scan rate, 0.060 mT/s.
(PDF)

Table S1 **Stereological analysis of oral tempol effects in the volume of spinal cord and of its anatomical regions.**
(PDF)
