# Supplementary material for: Tempol Moderately Extends Survival in a hSOD1G93A ALS Rat Model by Inhibiting Neuronal Cell Loss, Oxidative Damage and Levels of Non-Native hSOD1G93A Forms
Source: PLoS One. 2013 Feb 6;8(2):e55868. doi: 10.1371/journal.pone.0055868 (PMC3566093; doi:10.1371/journal.pone.0055868)
Supplement: Figure S1 — Representative EPR spectra of tissues of G93A rats treated with i.p. tempol. The animals received tempol i.p. (26 mg/rat) and were euthanized at the different times specified in the figure. The spectrum of each sample was scanned before and after the addition of 1 mM ferricyanide. The shown spectra are representative of the spectra obtained from homogenates of 3 different G93A rats (pre-symptomatic phase) treated with i.p. tempol. Instrumental conditions: microwave power, 10 mW; modulation amplitude, 0.1 mT; time constant, 163 ms; scan rate, 0.060 mT/s. (PDF) [file pone.0055868.s001.pdf]

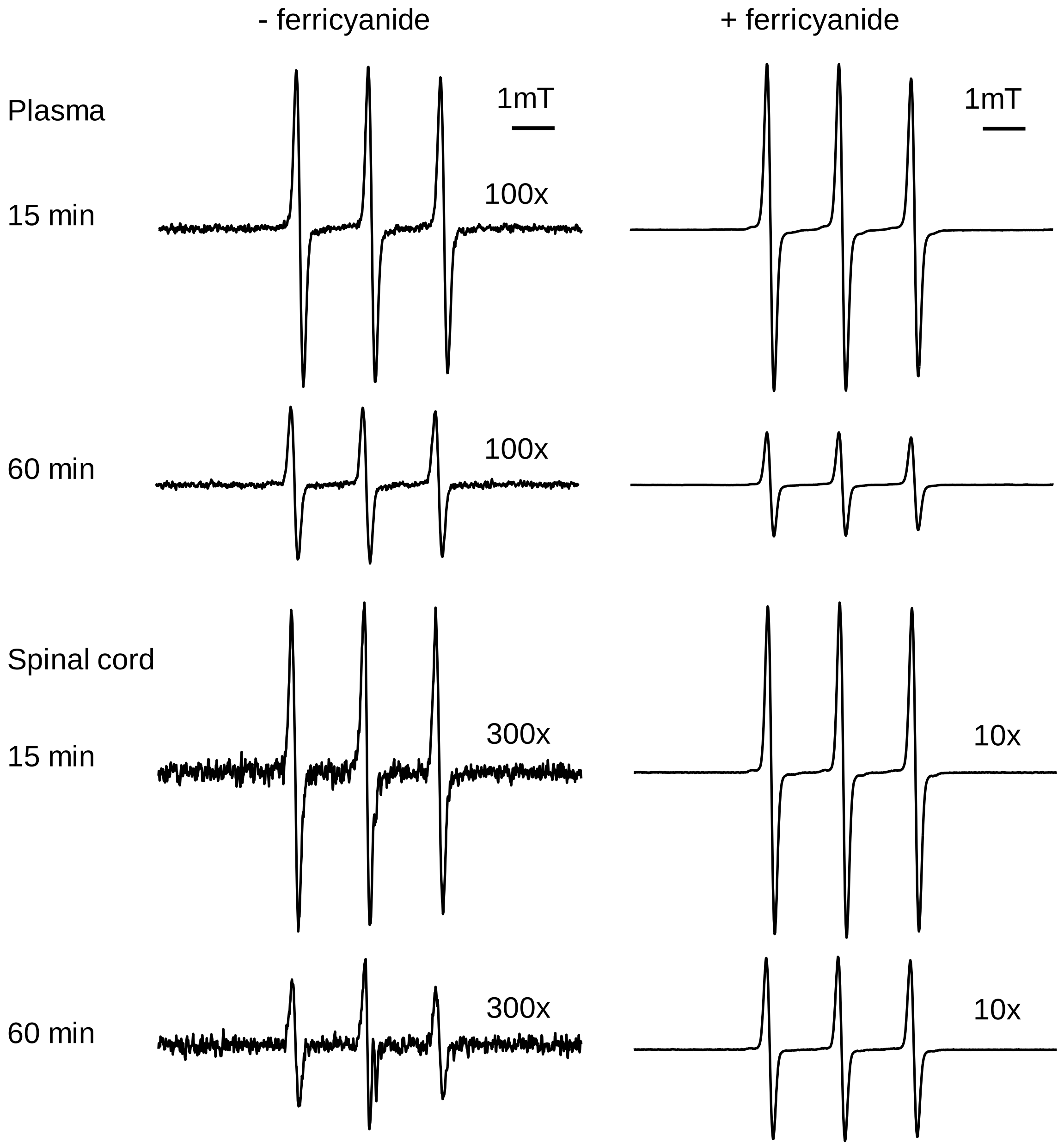

Figure S1. Representative EPR spectra of tissues of G93A rats treated with i.p. tempol. The animals received tempol i.p. (26 mg/rat) and were euthanized at the different times specified in the figure. The spectrum of each sample was scanned before and after the addition of 1 mM ferricyanide. Instrumental conditions: microwave power, 10 mW; modulation amplitude, 0.1 mT; time constant, 163 ms; scan rate, 0.060 mT/s.
