# Supplementary material for: Tempol Moderately Extends Survival in a hSOD1G93A ALS Rat Model by Inhibiting Neuronal Cell Loss, Oxidative Damage and Levels of Non-Native hSOD1G93A Forms
Source: PLoS One. 2013 Feb 6;8(2):e55868. doi: 10.1371/journal.pone.0055868 (PMC3566093; doi:10.1371/journal.pone.0055868)
Supplement: Table S1 — Stereological analysis of oral tempol effects in the volume of spinal cord and of its anatomical regions. (PDF) [file pone.0055868.s002.pdf]

**Table S1**. Stereological analysis of oral tempol effects in the volume of spinal cord anatomical regions.

| Anatomical region | Wild-type Volume ($mm^3$) | Untreated G93A | Treated G93A |
|---|---|---|---|
| **lumbar spinal cord**[NS1] | 45.40 (0.53)[a] | 55.22 (0.15)[a] | 55.80 (0.25)[a] |
| **white matter**[NS2] | 19.10 (0.54)[a] | 25.71 (0.16)[a] | 24.74 (0.28)[a] |
| **grey matter** [NS3] | 26.26 (0.52)[a] | 29.50 (0.14)[a] | 31.05 (0.22)[a] |
| **ventral horn**[NS4] | 11.28 (0.46)[a] | 12.71 (0.16)[a] | 13.08 (0.17)[a] |

Stereological estimates for volume ($mm^3$) of lumbar spinal cord and its compartments (white matter, grey matter and ventral horn) from G93A rats untreated and treated with tempol (30mM) in drinking water. Values are group means (CVs). Means that share a letter are not significantly different ([NS1]$p=0.704$; [NS2]$p= 0.545$; [NS3]$p=0.814$; [NS4]$p=0.803$).
